# Legal Frameworks for Governing Generalist and Agentic Artificial Intelligence in Medical Imaging

**DOI:** 10.7759/cureus.117090

**Published:** 2026-09-28

**Authors:** Pradosh Kumar Sarangi, Suvrankar Datta

**Affiliations:** 1 Radiodiagnosis, All India Institute of Medical Sciences, Deoghar, IND; 2 Radiology, Koita Centre for Digital Health, Ashoka University, Sonepat, IND

**Keywords:** accountability, agentic artificial intelligence, generalist artificial intelligence, large language models (llm), medical imaging

## Abstract

Artificial intelligence (AI) in medical imaging is moving from narrow algorithms toward generalist systems that perform multiple tasks and agentic workflows that plan, invoke tools or specialist models, and initiate downstream actions. This transition exposes a mismatch between what current AI and medical-device regulation commonly evaluates: a defined product with an intended use and the variable human-AI workflows that increasingly produce clinical consequences. We propose a five-layer accountability framework spanning data, model, orchestration, clinical use, and institutional lifecycle to demonstrate how familiar legal and governance principles can be translated into auditable controls. Clinically consequential uses will initially require meaningful clinician oversight, but this should not make the clinician the only, or even the default, bearer of responsibility for all upstream failures. Generalist and agentic AI systems may eventually introduce a varying amount of autonomy in healthcare systems, requiring us to rethink existing frameworks of clinical AI governance, with institutions, developers, and insurers requiring new role-specific obligations and coverage models calibrated to the degree of autonomy across the full decision pathway.

## Editorial

Medical imaging was among the earliest clinical domains to adopt AI because many tasks could be framed as bounded and verifiable, including detecting a nodule, segmenting a tumor to quantify its volume, or prioritizing a worklist as per standard risk scores. The corresponding legal and regulatory requirements have since evolved, but that frame is now becoming incomplete with the advent of generalist multimodal systems that can accept heterogeneous images and clinical text, switch among classification, segmentation, and report generation, and coordinate several capabilities within one system. A recent generalist model, MedVersa, exemplifies this transition by using a language model as an orchestrator for diverse imaging tasks [1]. Agentic workflows extend this transition further by enabling systems to retrieve professional guidelines, invoke diverse tools or AI models, compare intermediate outputs, draft a report, or trigger downstream processes with varying degrees of human intervention [2]. Generalist and agentic AI systems are related but distinct: generalist refers to breadth across tasks or modalities, whereas agentic refers to the capacity to plan and act across multiple steps. The increasing performance of these systems has started challenging the assumption that an AI system should continue to perform only one specific task and produce a single bounded output.

When a clinical consequence emerges from a chain of data sources, models, prompts, retrieval systems, tool calls, interface choices, and clinician decisions, the legally relevant unit is now shifting from the algorithm or clinician alone to the configured deployment of AI systems: the models and versions in use; the tasks they are permitted to perform; the information they can access; the actions they can initiate; the way outputs are shown to clinicians; and the institution's arrangements for monitoring and intervention. Current medical-device regulation, data-protection law, professional-negligence doctrine, and institutional governance each capture only part of this system.

While no single international legal framework comprehensively governs this transition today, with the precise allocation of responsibility remaining jurisdiction-specific, the principles underlying those rules are nevertheless more transferable. Across jurisdictions and professional frameworks, legal and governance discussions repeatedly invoke known concepts like duty of care, foreseeability, causation, proportionality, traceability, non-discrimination, and contestability. FUTURE-AI similarly places trustworthy healthcare AI within a lifecycle framework, and the practical challenge now is to move from articulating principles to producing evidence that they were respected in a particular deployment [3].

We propose a five-layer accountability framework for generalist and agentic AI systems in medical imaging to identify where control resides and what evidence should be available when a system is authorized, audited, challenged, or implicated in patient harm. The framework spans data, model, orchestration, clinical, and institutional and lifecycle accountability; although a failure may cross several layers, responsibility should not disappear between them (Figure 1).

**Figure 1:**
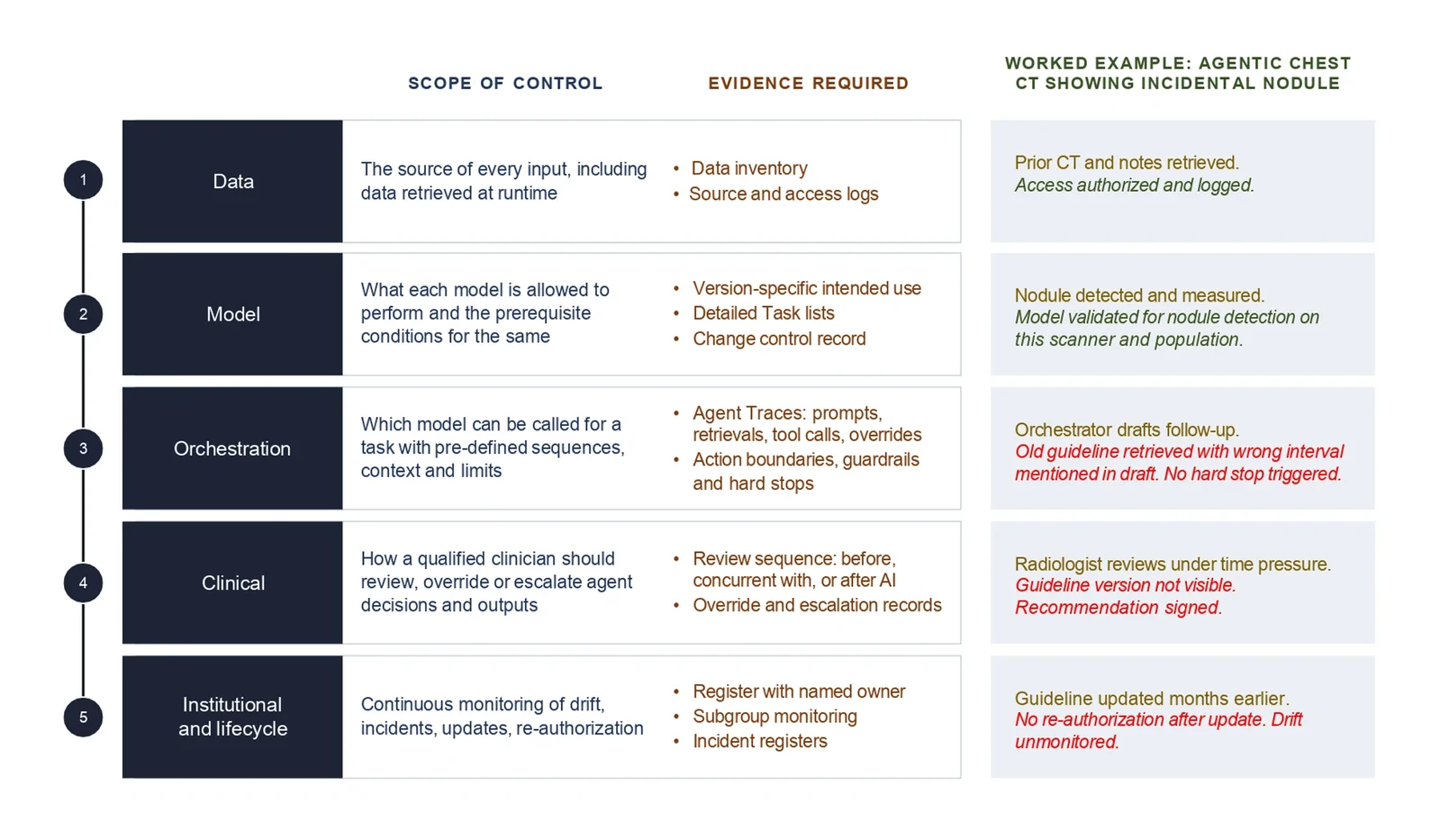
The five-layer accountability framework applied to an agentic chest CT workflow Each row demonstrates one accountability layer, with the decisions made at that layer and the evidence any agentic deployment should be able to produce. The column on the right follows a hypothetical incidental pulmonary nodule case in which the orchestrator accidentally retrieves a superseded follow-up guideline. Green indicates a control that operated as intended and red a control that failed or did not detect the error. Responsibility as shown is distributed across layers. Image credits: Suvrankar Datta. The figure was prepared using Microsoft PowerPoint (Microsoft Corporation, Redmond, WA, USA). Abbreviations: AI, artificial intelligence; CT, computed tomography

Data accountability begins with a simple question asking where each input in the workflow comes from and whether that is allowed by the system’s predefined guardrails. An agentic system can retrieve a defined corpus of clinical records and access external knowledge sources at runtime. An institution must be able to control the access and assess whether the purpose for which the data were retrieved was pre-authorized. The data inventories, source records, and access logs for each agentic run must be stored for future audits.

Model accountability concerns the tasks each model in the system is allowed to perform and the conditions under which the authorization remains valid. Every authorized function requires a version-specific statement of intended use, prior evidence of performance in the relevant population where it is being deployed, and disclosure of all failure modes. Average task accuracy is insufficient when a generalist system can perform multiple tasks. Evaluation of such systems must be on different individual tasks with clinically relevant subgroup performance guarantees. Local validation and uncertainty assessment must be a prerequisite with documented change control plans.

Orchestration accountability and benchmarking is often overlooked by current model-focused regulations but is becoming increasingly important. The orchestrator may typically be a large language model (LLM) or vision language model (VLM) that selects which model or tool to invoke, decides what context to retrieve, plans the sequence, and judges when the run needs to be repeated. An agentic deployment requires specific boundaries, access controls, and predefined guardrails for high-risk tasks with clinician-validated handover rules. An execution trace must record all prompts, retrieved sources, tool calls, and intermediate outputs. Recent proposals like MedLog for event-level logging demonstrate what this looks like in practice, recording each AI invocation and supporting more detailed traces for agentic workflows, offering granular insights into agent actions [4].

Clinical accountability and the extent to which it is required in autonomous workflows is going to evolve significantly with time, but for the near future, we believe that clinically consequential outputs from generalist and agentic systems must remain subject to meaningful oversight by a qualified clinician. However, just having a "human in the loop" does not guarantee true oversight if the clinician lacks sufficient information, time, and competence to review the output and the authority to override, escalate, or reject the AI recommendation. Each clinical deployment should specify whether the radiologist interprets independently before seeing the AI output or reviews it concurrently because the sequence in which a human expert and AI collaborate should be chosen as per the available evidence.

Institutional and lifecycle accountability covers decisions which lie beyond any individual radiologist's control. The procurement, integration, staff training, performance monitoring, updates, and rollback decisions for an autonomous workflow are often decided by the institution. The deploying institution must maintain deployment registers, name an accountable owner, monitor audit logs and performance regularly, and reauthorize the system after new changes. Developers should also provide versioned documentation and mechanisms to investigate reproducible failures. How intensively this needs to be done should depend on the clinical consequence and scale with increasing autonomy.

The accountability framework is not for proposing a new legal doctrine but to make existing responsibilities visible. A deployment should make clear who was responsible for review and escalation, which foreseeable risks were tested, how the decision pathway can be reconstructed, and whether safeguards were proportionate to the system's clinical consequence and degree of autonomy. It should also preserve practical routes to override or correct an AI-mediated decision and to report and investigate failures. These controls are necessary to make the facts needed for regulatory, institutional, and judicial scrutiny available.

Accountability must also be measurable, and depending on the use case, relevant evidence may include data-provenance completeness, unauthorized-access events, task-specific and subgroup performance, version-to-version regressions, orchestration and handover failures, clinician override and escalation patterns, incident-detection latency, corrective-action time, and rollback performance. These are illustrative indicators, and our central argument is that a clinically consequential deployment must demonstrate whether its safeguards are functioning as expected.

The continued need for clinician oversight, as we have in the majority of AI deployments today, does not mean that every injury should be treated as a clinician's error. We argue for role-specific responsibility proportionate to control over the relevant risk, representations made about the system, and capacity to prevent harm. Clinicians should remain accountable for judgments they were realistically equipped and expected to make; institutions for selection, integration, staffing, training, monitoring, and continued deployment; and developers or manufacturers for design defects, misleading claims, inadequate warnings, cybersecurity failures, and unsafe updates [5].

Insurance will also have to evolve alongside liability. Conventional medical-indemnity and product-liability policies each cover part of an AI-mediated event but leave uncertainty at their boundaries. A more coherent approach would be to underwrite the configured workflow according to the authority delegated to AI and the residual capacity for human intervention. Assistive, collaborative, and autonomous workflows should not carry identical premiums, exclusions, or evidentiary requirements. Relevant underwriting variables must include the authority delegated to AI, whether case-level clinician review remains mandatory, the clinician's practical ability to intervene in all cases, system observability, and the clinical consequence of an erroneous action. Insurers could also condition coverage on local validation, auditable execution records, explicit handover criteria, continuous monitoring, incident reporting, and independent review. If properly designed with inputs from clinical AI experts, we envision that such underwriting would make insurance a prospective governance lever rather than only a mechanism for compensation after harm. We must be cautious, however, to ensure that the new paradigm does not make coverage inaccessible in resource-constrained settings, encourage defensive overregulation, or allow insurance to substitute for safety.

Conclusion

As generalist and agentic AI promise to make medical imaging more accessible, the same advantages that make these systems useful, such as their ability to do multiple tasks and act through networks of components, also complicate the legal accountability of such systems. These systems cannot be governed like traditional static and standalone algorithms, nor should placing a clinician at the end of the chain be treated as a complete answer to accountability. New legal frameworks need to be urgently developed for governing the full human-AI pathway through which clinical consequences are produced, and we believe that our accountability framework will guide jurisdictions for developing appropriate governance structures for such systems.
